# Coccidioidal Meningitis in an Immunocompetent Patient: A Rare Central Nervous System Infection

**DOI:** 10.7759/cureus.111427

**Published:** 2026-06-24

**Authors:** Norlando José Chávez Durón, Lorenzo E Aragón Conrado, Francgiliz J Robleto, Ledixa Zeledon, Maria Gabriela Zelaya Aróztegui

**Affiliations:** 1 Internal Medicine/Infectious Diseases, Hospital Escuela Oscar Danilo Rosales Arguello, Universidad Nacional Autónoma de Nicaragua, Leon, NIC; 2 Medical Education, Escuela de Medicina Teniente Coronel y Doctor Sergio Martinez Ordoñez, Managua, NIC; 3 Medical Education, Hospital Militar Escuela Dr. Alejandro Davila Bolaños, Managua, NIC; 4 Internal Medicine, Hospital Militar Escuela Dr. Alejandro Davila Bolaños, Managua, NIC; 5 Obstetrics and Gynaecology, Universidad Nacional Autónoma de Nicaragua, Leon, NIC; 6 Internal Medicine, Universidad Nacional Autónoma de Nicaragua, Leon, NIC

**Keywords:** amphotericin b, diabetic ketoacidosis (dka), emergency surgical debridement, rhino-orbital-cerebral mucormycosis (rocm), rhizopus

## Abstract

Coccidioidomycosis is a systemic fungal infection caused by *Coccidioides immitis *and *Coccidioides posadasii*. Although well recognized in parts of the southwestern United States, it remains underdiagnosed in Mexico and Central America. While most cases present as self-limited pulmonary infection, approximately 1% of immunocompetent individuals develop disseminated disease, with coccidioidal meningitis (CM) being the most severe manifestation. We describe a 46-year-old immunocompetent male from Oaxaca, Mexico, who presented with progressive headache, neurocognitive decline, and altered mental status. Initial cerebrospinal fluid (CSF) analysis suggested cryptococcal meningitis, yet cryptococcal antigen testing and fungal cultures were negative. Empirical antifungal therapy produced only partial improvement. Given his occupational exposure in Texas and evolving clinical course, coccidioidomycosis was suspected. Serologic testing (enzyme immunoassay, EIA) and a coccidioidin skin test were positive, and prolonged CSF culture ultimately yielded *Coccidioides *spp., confirming CM. High-dose fluconazole led to progressive neurologic recovery, and the patient remains asymptomatic on maintenance therapy. This case highlights the diagnostic challenges of CM in regions where coccidioidomycosis is not traditionally considered endemic. Non-specific presentations and initial negative studies may delay diagnosis, underscoring the importance of epidemiologic suspicion, repeated testing, and prolonged fungal culture. Early recognition and timely initiation of azole therapy are essential to prevent the high morbidity and mortality associated with this condition.

## Introduction

Coccidioidomycosis is a systemic fungal infection caused by *Coccidioides immitis* and *Coccidioides posadasii*, dimorphic fungi that inhabit desert soils. The disease is endemic in the southwestern United States, particularly Southern California's San Joaquin Valley, where it is well characterized, and in Mexico, as well as scattered areas of Central and South America, where it remains underrecognized [1]. Most infections present as self-limited pneumonia, but approximately 1% of immunocompetent individuals develop dissemination with predilection for soft tissue, joints, and the central nervous system.

Coccidioidal meningitis (CM) is the most severe extrapulmonary manifestation, associated with high morbidity and mortality if untreated [2]. Diagnosis is frequently delayed due to its nonspecific presentation and resemblance to other types of meningitis, particularly in regions where the disease is underrecognized [3]. We report a case of CM in an immunocompetent patient from Mexico to highlight diagnostic challenges and the importance of epidemiological suspicion in both endemic and nontraditional regions.

## Case presentation

A 46-year-old male from Pochutla, Oaxaca, Mexico, employed as a heavy machinery operator in the United States (Texas), with a history of recently diagnosed type 2 diabetes mellitus, arterial hypertension, and chronic alcohol use (biweekly intake to the point of intoxication since the age of 18).

Three weeks before admission, he developed a progressive, oppressive frontal headache of insidious onset, exacerbated by exertion and without relieving factors. This was followed by nausea, emesis, blurred vision, hyporexia, and generalized weakness, which progressively impaired his daily activities. Despite multiple hospital visits, treatment with parenteral hydration and analgesics did not result in improvement.

Five days before admission, he developed somnolence, incoherent speech, disorientation, erratic behavior, and intermittent unresponsiveness. Two days before admission, he became stuporous and was admitted to the Regional Hospital of Zumpango with suspected neuroinfection. Lumbar puncture revealed cerebrospinal fluid (CSF) with chloride of 102 mmol/L, protein of 177.3 mg/dL, glucose of 55 mg/dL (CSF-to-serum ratio: 0.25), density of 1.02, clear appearance, and a positive India ink stain, raising the suspicion of CM (Table 1). Due to the unavailability of amphotericin B, he was transferred to the Hospital General de México.

**Table 1:** Cerebrospinal fluid analysis Initial cerebrospinal fluid (CSF) analysis performed at the Regional Hospital of Zumpango. Reference ranges represent standard adult laboratory values. Abbreviations: CSF, cerebrospinal fluid; mg/dL, milligrams per deciliter; mmol/L, millimoles per liter

Parameter	Patient Value	Reference Range	Units
CSF appearance	Clear	Clear	-
CSF glucose	55	40-70	mg/dL
CSF-to-serum glucose ratio	0.25	>0.6	Ratio
CSF protein	177.3	15-45	mg/dL
CSF chloride	102	118-132	mmol/L
CSF density	1.02	1.005-1.030	g/mL
India ink stain	Positive	Negative	-

**Figure 1:**
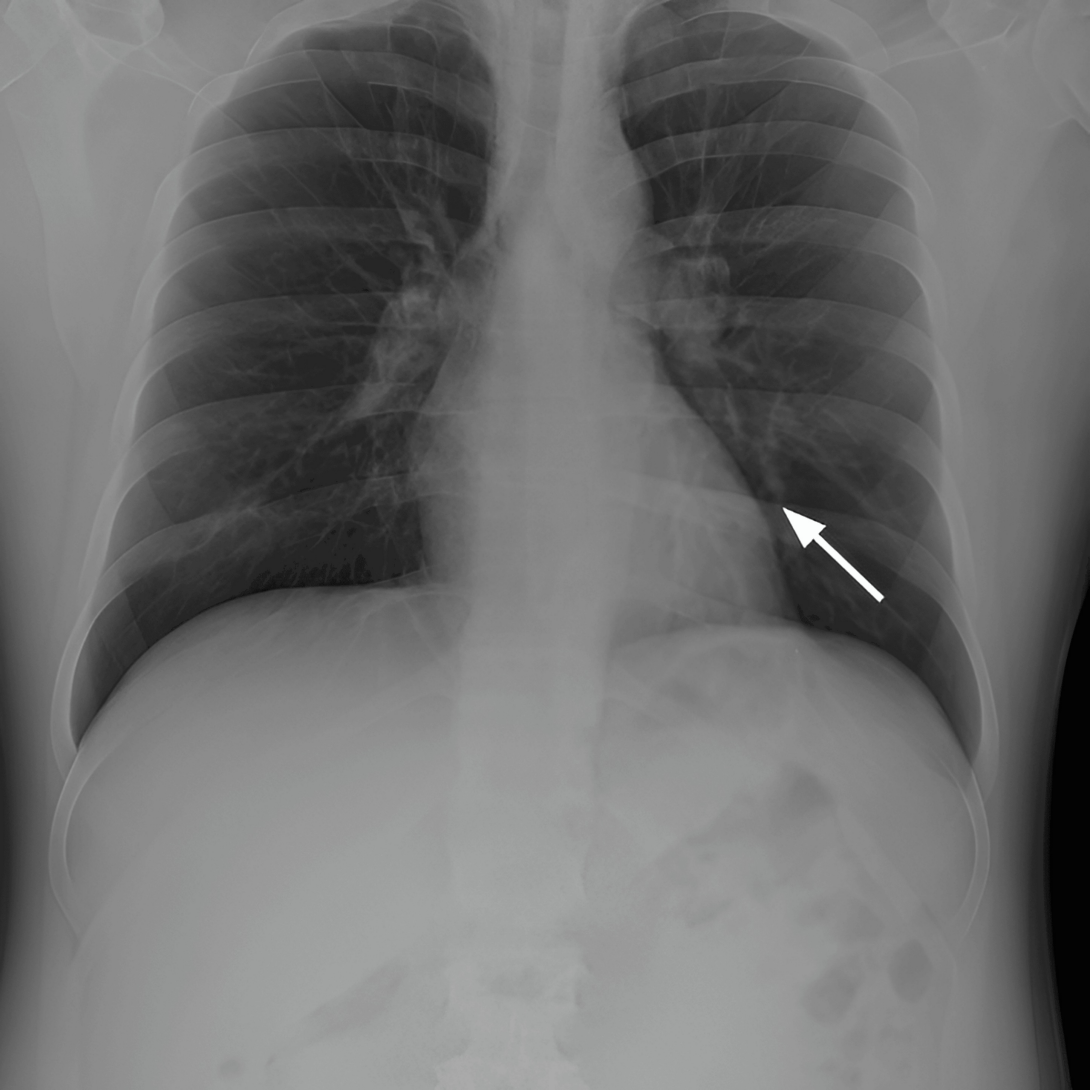
Posteroanterior chest radiograph Posteroanterior chest radiograph demonstrating right parahilar lymphadenopathy (white arrow), consistent with mediastinal involvement. This radiographic finding supports pulmonary involvement in disseminated coccidioidomycosis.

On admission, neurological examination showed a Glasgow Coma Scale score of 14 (O4, V4, M6), fluent but incoherent language, disorientation in time, place, and circumstance, and episodic memory impairment with mild emotional lability [4]. No cranial nerve abnormalities, meningeal signs, or features of intracranial hypertension were noted. Subsequently, he developed psychomotor agitation. A repeat lumbar puncture revealed turbid CSF with an opening pressure of 22 cm H₂O, leukocytes of 0/mm³, crenocytes of 11/mm³, glucose of 129 mg/dL, protein of 153.16 mg/dL, lactate dehydrogenase (LDH) of 15 U/L, and chloride of 124.5 mmol/L (Table 2). Chest radiography demonstrated right parahilar lymphadenopathy (Figure 1).

**Table 2:** Repeat cerebrospinal fluid analysis Repeat cerebrospinal fluid (CSF) analysis performed at the Hospital General de México. Reference ranges represent the standard adult laboratory values. Abbreviations: CSF, cerebrospinal fluid; LDH, lactate dehydrogenase; cm H₂O, centimeters of water; mg/dL, milligrams per deciliter; mmol/L, millimoles per liter

Parameter	Patient Value	Reference Range	Units
Opening pressure	22	6-20	cm H₂O
CSF appearance	Turbid	Clear	-
CSF leukocytes	0	0-5	cells/mm³
Crenocytes	11	0	cells/mm³
CSF glucose	129	40-70	mg/dL
CSF protein	153.16	15-45	mg/dL
CSF chloride	124.5	118-132	mmol/L
Lactate dehydrogenase (LDH)	15	10-40	U/L

**Figure 2:**
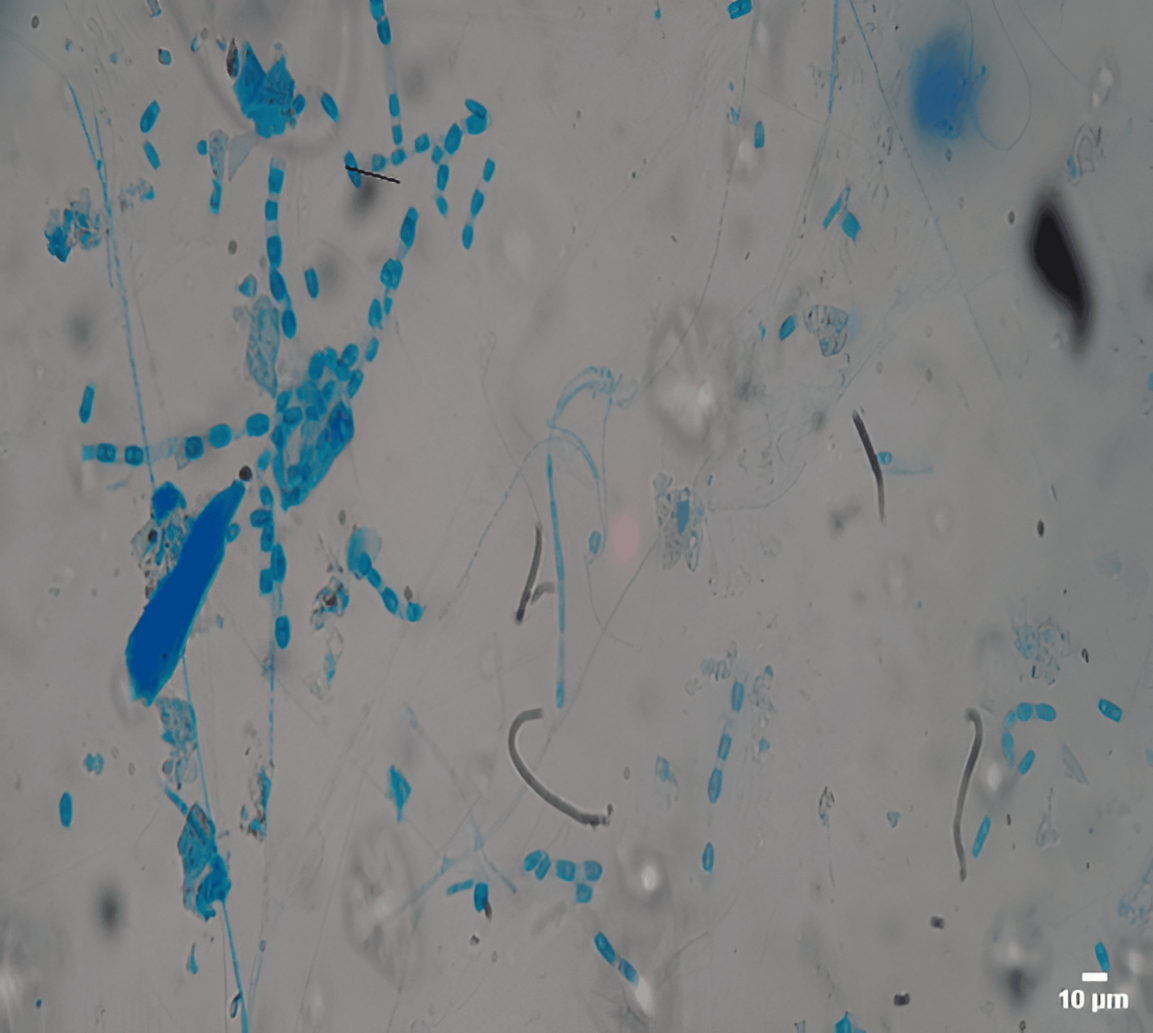
Microscopic appearance of Coccidioides spp. isolated from cerebrospinal fluid culture Microscopic appearance of *Coccidioides *spp. isolated from cerebrospinal fluid culture, showing characteristic spherules containing endospores consistent with the parasitic phase of the organism. Lactophenol cotton blue stain, 400× magnification. The scale bar represents 10 µm.

Empirical therapy with amphotericin B deoxycholate plus fluconazole was initiated, with only partial clinical improvement. Bronchoscopy was performed, and bronchoalveolar lavage samples were sent for fungal culture, which yielded negative results; CSF fungal cultures were also negative. CSF cryptococcal antigen was negative. Given the epidemiological background and clinical presentation, coccidioidomycosis was suspected. Coccidioidal antibody testing by enzyme immunoassay (EIA; sensitivity of 65%, specificity of 98%) and the coccidioidin skin test were both positive. A subsequent CSF culture with prolonged incubation demonstrated growth of *Coccidioides *species (Figure 2). The patient was transitioned to high-dose fluconazole, with progressive clinical improvement. At present, he remains on fluconazole of 400 mg every 12 hours, has responded satisfactorily to treatment, and is asymptomatic with no neurological deficits.

## Discussion

Coccidioidomycosis (Valley fever) is an infectious disease caused by dimorphic fungi belonging to the genus *Coccidioides *[5]. *Coccidioides *was originally described as one species, *C. immitis*. Genetic studies have identified two distinct *Coccidioides *species with different geographic distributions. *C. immitis *is primarily found in California and parts of Eastern Washington, while *C. posadasii *accounts for nearly all cases in Texas, Central, and South America. Despite these genetic distinctions, no significant differences in clinical presentation, pathogenicity, or disease progression have been observed between the two species [6].

*Coccidioides *is a dimorphic fungus that alternates between saprobic (mycelia) and parasitic (arthroconidia/spherule/endospore) phases [5]. Heightened risk is linked to outdoor occupational or recreational exposure. Fluctuations in incidence are influenced by intermittent seasonal precipitation, the intensity of wind-driven dust storms, sustained immigration of immunologically naïve hosts to endemic areas, and mechanical disruption of soil surfaces (with subsequent aerosolization of fungal spores) due to construction projects, wildfires, or seismic events [7].

Infection is almost always acquired through inhalation of a single arthroconidium, as its small size helps it reach the terminal bronchioles and alveoli. Within the lungs, an arthroconidium transforms from a barrel-shaped cell into a spherical structure, which subsequently enlarges considerably, sometimes reaching a diameter of 70 microns or more. These enlarged spherules develop internal septations, and within each subcompartment, individual cells (endospores) evolve [8].

The organism's pathogenicity lies in the resistance of spherules to phagocytosis, leading to persistent suppuration and necrosis. Neutrophils and macrophages are recruited but fail to eradicate the fungus, making T-cell-mediated immunity essential for control. Dissemination occurs when fungal elements spread through lymphatic and hematogenous routes, often in immunocompromised patients, the elderly, or late pregnancy, with impaired cell-mediated immunity contributing to disease progression [2].

It is estimated that fewer than half of all infections receive medical attention, as the disease is often subclinical. Among patients who develop symptoms, a wide spectrum of clinical manifestations is possible. Primary infection most frequently presents as community-acquired pneumonia approximately 7-21 days after exposure. Patients may experience constitutional symptoms such as fever, night sweats, weight loss, profound fatigue, and arthralgias that can significantly interfere with activities of daily living and may persist for several months. The frequent complaint of arthralgia has contributed to the alternative term "desert rheumatism" for this disease [9].

Only about 1% of immunocompetent patients will develop extrapulmonary infection. Months after pulmonary involvement, *C. immitis *may disseminate to the central nervous system, where it can cause several neurological syndromes, including CM [9]. This is the most devastating complication, and if left untreated, it is universally fatal. It may copresent with primary disease, appearing some weeks or months after primary infections or in persons with no notable primary infection [6]. Infectious vasculitis, hydrocephalus, and cerebral infarctions are recognized complications of fungal meningitis in these patients. Hydrocephalus, in particular, occurs in up to 50% of cases and increases the risk of mortality by up to 12-fold [10].

The diagnosis of CM begins with including it in the differential diagnosis, encouraged by the clinical presentation aforementioned. Focal neurologic deficits, particularly visual disturbances, can signal severe CNS involvement. Neuroimaging, ideally with gadolinium-enhanced MRI, can help support the diagnosis. About half of patients show abnormalities such as hydrocephalus, basilar meningeal enhancement, basilar vasculitis infarction, or, less commonly, an abscess or mass lesion [11].

In cases of early infection, repeated serological testing or attempts to directly visualize or culture the organism may be the only means of establishing the diagnosis. Since Coccidioides is never part of the normal microbiota, its identification in respiratory secretions, tissues, or other patient specimens-whether by direct examination or by culture-constitutes definitive evidence of infection, and in this case, a CSF culture positive for *Coccidioides *species is diagnostic of CM.

Coccidioidal antigen can also be detected in urine, serum, and other body fluids. This test may be particularly useful in severely immunocompromised hosts with negative serology and in those with extensive disease. It can also aid in the diagnosis of CM. The US Food and Drug Administration has approved a reformulated skin test for coccidioidomycosis (Spherusol, Nielsen BioSciences, San Diego, CA) to evaluate delayed-type hypersensitivity responses in patients previously diagnosed with pulmonary coccidioidomycosis. Although not approved for screening, its use for this purpose has been supported by published studies employing spherulin and other coccidioidal skin test preparations [12,13].

In patients with CM, guidelines recommend high-dose fluconazole or itraconazole. Amphotericin B is currently reserved for the treatment of refractory cases because it is highly nephrotoxic. It is also the drug of choice during the first trimester of pregnancy. In general, immunocompetent patients require treatment for three to six months; however, in patients with underlying conditions leading to immunosuppression, as well as those with CM who improve or become asymptomatic, therapy should be continued indefinitely [13].

## Conclusions

CM remains a life-threatening but underrecognized entity outside classic endemic regions. This case illustrates the importance of maintaining a high index of suspicion in patients with compatible clinical syndromes and a history of residence or occupational exposure in areas where *Coccidioides *species are present. Diagnostic delays are common due to nonspecific symptoms and initially negative serologic or microbiologic results, making repeated testing and prolonged culture essential. Early initiation of high-dose azole therapy is critical for improving outcomes, as CM is uniformly fatal without treatment. Strengthening awareness among clinicians in nontraditional regions is key to enabling timely diagnosis and reducing morbidity and mortality.
